# Technical and legal aspects of federated learning in bioinformatics: applications, challenges and opportunities

**DOI:** 10.3389/fdgth.2025.1644291

**Published:** 2025-11-18

**Authors:** Daniele Malpetti, Marco Scutari, Francesco Gualdi, Jessica van Setten, Sander van der Laan, Saskia Haitjema, Aaron Mark Lee, Isabelle Hering, Francesca Mangili

**Affiliations:** 1 Istituto Dalle Molle di Studi sull’Intelligenza Artificiale (IDSIA), USI-SUPSI, Polo Universitario Lugano, Lugano, Switzerland; 2 Swiss Institute of Bioinformatics (SIB), Lugano, Switzerland; 3 Department of Cardiology, University Medical Center Utrecht, University of Utrecht, Utrecht, Netherlands; 4 Central Diagnostics Laboratory, University Medical Center Utrecht, University of Utrecht, Utrecht, Netherlands; 5 Department of Genome Sciences, University of Virginia, Charlottesville, VA, United States; 6 William Harvey Research Institute, NIHR Barts Biomedical Research Centre, Queen Mary University of London, London, United Kingdom; 7 Étude Hering, DPO Associates SARL, Nyon, Switzerland

**Keywords:** federated machine learning, exposome, secure distributed analysis, data privacy, collaborative genomics

## Abstract

Federated learning leverages data across institutions to improve clinical discovery while complying with data-sharing restrictions and protecting patient privacy. This paper provides a gentle introduction to this approach in bioinformatics, and is the first to review key applications in proteomics, genome-wide association studies (GWAS), single-cell and multi-omics studies in their legal as well as methodological and infrastructural challenges. As the evolution of biobanks in genetics and systems biology has proved, accessing more extensive and varied data pools leads to a faster and more robust exploration and translation of results. More widespread use of federated learning may have a similar impact in bioinformatics, allowing academic and clinical institutions to access many combinations of genotypic, phenotypic and environmental information that are undercovered or not included in existing biobanks.

## 1 Introduction

Sharing personal information has been increasingly regulated in both the EU [with the GDPR and the AI act; (1, 2)] and the US [with HIPAA and the National AI Initiative Act; (3, 4)] to mitigate the personal and societal risks associated with their use, particularly in connection with machine learning and AI models (5). These regulations make multi-centre studies and similar endeavours more challenging, impacting biomedical and clinical research.

Federated learning [FL; (6, 7)] is a technical solution intended to reduce the impact of these restrictions. FL allows multiple parties to collaboratively train a global machine learning model using their respective data without sharing it themselves, and without any meaningful model performance degradation. Instead, parties only share model updates, making it impractical to reconstruct personal information when the appropriate secure computational measures are implemented (8).

This approach strengthens *security* by keeping sensitive information local, improves *privacy* by minimising data exposure even between the parties involved, and limits *risk* of data misuse by allowing each party to retain complete control over its data (9). If enough parties are involved, FL may access larger and more varied data pools than centralised biobanks can provide. This is particularly true if there are legal (or other) barriers to data centralisation, resulting in more accurate and robust models than those produced by any individual party.

FL has proven to be a valuable tool for biomedical research and is expected to gain further traction in the years to come. Its use has improved breast density classification models [accuracy up by 6%, generalisability up by 46%; (10)], COVID-19 outcome prediction at both 24h and 72h [up 16% and 38%; (11)] and rare tumour segmentation [up by 23%–33% and 15%; (12)] compared to single-party analyses. A consortium of ten pharmaceutical companies found that FL improved structure-activity relationship (QSAR) models for drug discovery [both up 12% (13)]. Early-stage applications building predictive models from electronic health records (14) have also confirmed no practical performance degradation compared to pooling data from all parties.

To achieve such results, a real-world implementation of FL must overcome several methodological, infrastructural and legal issues. However, biomedical FL literature reviews (15, 16, among others) are predominantly high-level and considered simulated rather than real-world implementations. Here, we will cover federated methods designed explicitly for bioinformatics and discuss the infrastructure they require, as well as how they meet legal requirements. While various legal frameworks may apply depending on jurisdiction, we place particular emphasis on the European General Data Protection Regulation (GDPR). This focus reflects not only our EU-based perspective but also the GDPR’s comprehensive scope, stringent requirements, and influence as a global benchmark for data protection in research and technology. In reviewing the literature, we selected papers that study practical analysis problems (as opposed to proposing methodologies in the abstract) for proteomics, genome-wide association studies (GWAS), and single-cell and multi-omics data. We also considered papers that discuss their feasibility, trade-offs, and performance compared to centralised analyses, and were published after 2016. We used Google Scholar to find and retrieve them.

To this end, we have structured the remainder of the paper as follows: We first review the fundamental concepts and design decisions of FL in Section 2, including different topologies (Section 2.1), hardware and software (Section 2.2), data layouts in different parties (Sections 2.3 and 2.4), security (Section 2.5) and privacy concerns (Section 2.6). In Section 3, we contrast and compare bioinformatics FL methods for proteomics and differential expression (Section 3.1), genome-wide association studies (GWAS; Section 3.2), single-cell RNA sequencing (Section 3.3), multiomics (Section 3.4) and medical imaging (Section 3.5) applications. We conclude the section with notable examples of ready-to-use software tools (Section 3.6). Section 4 provides examples of federated operations common in bioinformatics. Finally, we discuss the legal implications of using FL (Section 5) before summarising our perspective in Section 7.

## 2 Federated learning

**Figure 1:**
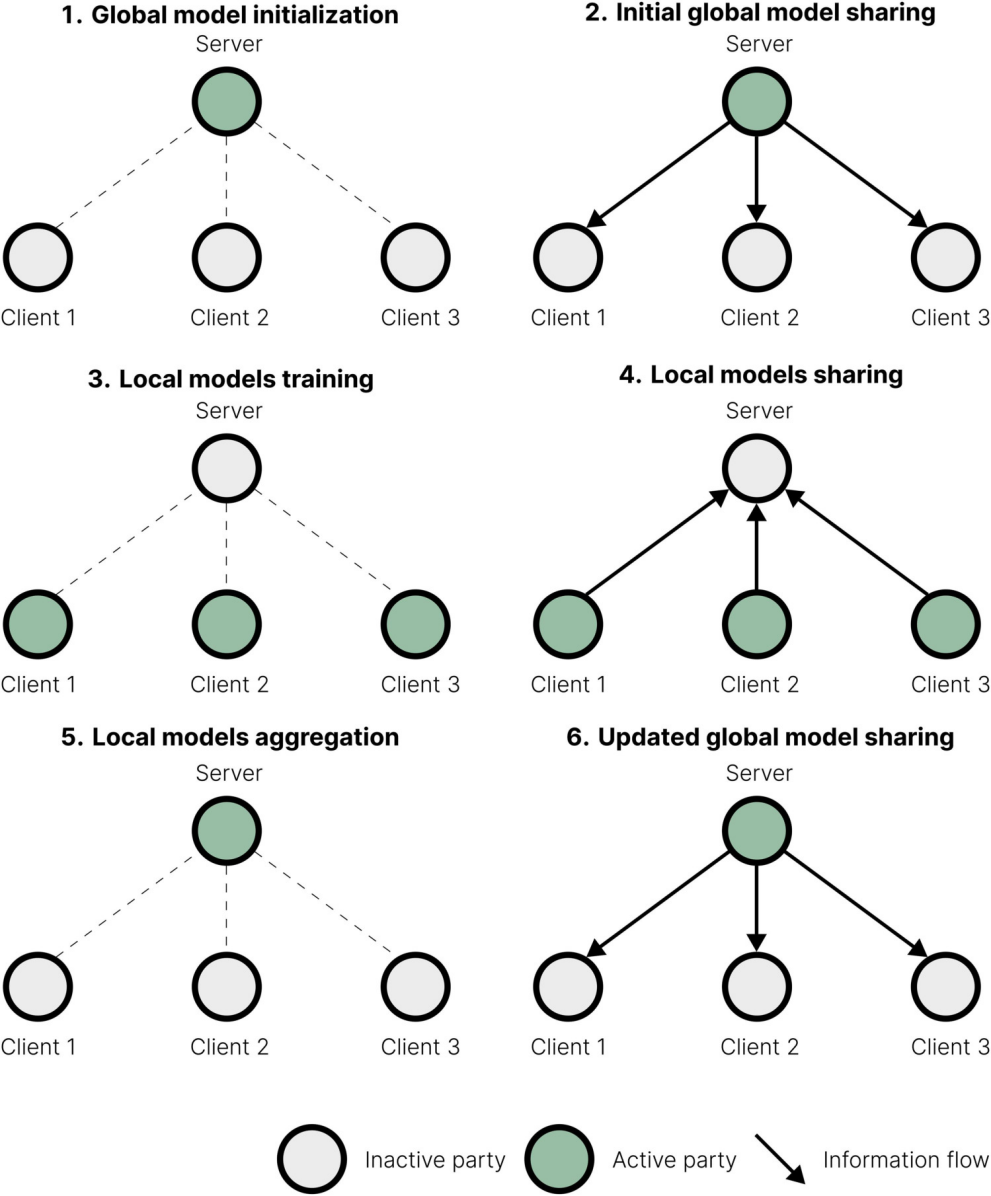
Overview of a typical federated learning (FL) workflow. (1) The central server initialises a *global model*. (2) The server shares the *global model* parameters with consortium parties, referred to as clients. (3) Each client initialises a *local model* from the global model parameters and updates it by training it on its local data. (4) Clients send their updated *local model* parameters back to the server. (5) The server aggregates local model parameters it collected to construct a new *global model*. (6) The server redistributes the updated *global model* parameters to clients to start the next training round. Steps (3)–(6) are repeated iteratively until a predefined stopping criterion is met. Active parties in each step are in green, and the arrows show the direction of information flow within the consortium.

FL is a collaborative approach to machine learning model training, where multiple institutions form a consortium to jointly train a shared model by exchanging model updates rather than individual patient data. Typically, FL involves data holders (called “clients”) sharing their local contributions with a server (6) as outlined in Figure 1. The server then creates and shares back a global model, inviting the data holders to update and resubmit their contributions. This process is iterative and involves several rounds of model update exchanges. Unlike traditional centralised computing, FL does not store patient data in a central location. Instead, patient data remain under the control of the respective data owners at their sites, enhancing privacy.

FL has similarities with *distributed computing*, *meta-analysis*, and *trusted research environments* (TREs), but also has key differences, which we highlight below. Table 1 provides a comparative overview of these approaches.

**Table 1:** Methodological comparison of centralised learning, federated learning, distributed computing, meta-analysis, and trusted research environments.

Aspect	Centralised learning	Federated learning	Distributed computing	Meta-analysis	Trusted research environments (TRE)
Primary goal	Aggregate all individual data into one place and train or analyse centrally.	Collaborative model training across parties without sharing individual data.	Increase speed and scalability; job parallelisation.	Combine evidence from completed studies.	Provide secure, auditable access to sensitive data for research.
Where individual data live	Single central repository.	Stay local at each device/institution.	Centrally stored, sharded across nodes.	Remain with original studies; not pooled.	In a secure environment or under local (federated) control (only relevant data are transferred).
How learning happens	Training/analysis is run on pooled data in one environment.	Participants compute local model updates and send them for secure aggregation in iterative rounds.	Tasks are partitioned and executed in parallel; results are combined centrally.	Study-level results are aggregated.	Researchers run code/queries inside the TRE; outputs are checked before release.
Participation	All data contributors must share data with the central site beforehand.	Multiple data holders, dynamic participation possible (devices can join/leave).	Centrally managed workers/nodes with data partitions.	Fixed set of completed/published studies.	Approved users/projects with strict governance and access control.
Data assumptions	No inherent assumption; depends on chosen analysis method.	Must handle non-IID data and uneven sample sizes.	Often assumes roughly IID, evenly partitioned data.	Models between-study heterogeneity (fixed/random effects).	No inherent assumption; depends on chosen analysis method.
What moves across parties	Individual data sent to the central site.	Model updates (gradients/weights), possibly in shares (SMPC), encrypted or differentially private.	Data blocks and intermediate results.	Study-level summary statistics.	Code/queries go in; vetted results come out.
Privacy posture	Highest data exposure (requires trust in central data custodian).	Designed to avoid individual data sharing; can support privacy-enhancing techniques.	Not privacy-focused (single trust domain).	Only summary results shared.	Via technical or organisational controls.
Output artefact	Single trained model or analysis result from pooled data.	Global or personalised model held by each participant.	Finished job outputs.	Summary results with uncertainty estimates.	Analysis outputs are released after disclosure control.
Typical examples	Central data warehouse, pooled data in a multi-centre study.	Cross-hospital FL; edge device FL.	Spark, Hadoop, Ray, HPC clusters.	Cochrane-style meta-analyses.	UK Biobank RAP, Federated EGA.
Legal responsibilities	The central data controller has the responsibility for legal compliance and security.	Data controllers retain responsibility for legal compliance and security; data processors have contractual responsibilities linked to that.	Depends on data origin: same as centralised learning for single-centre studies, or as federated learning when data comes from multiple centres.	Data controllers retain responsibility; data processors must ensure original data use agreements permit meta-analysis.	The operator is responsible for TRE security and governance. Data controllers retain legal responsibility for sharing the data.
Legal basis in addition to data subjects’ consent	Only data subjects’ informed consent is needed.	Data sharing agreements for pseudo-anonymised data.	Data sharing agreement for individual data in case data from multiple centres are aggregated.	No personal data involved if the data are sufficiently aggregated (anonymised); otherwise, same as federated learning.	Access agreements between TREs and data controllers: permitted uses, audit, and security protocols. Data processors’ agreements with TRE.

Consent from data subjects is assumed for data use.

Distributed computing (DC) (17) divides a computational task among multiple machines to enhance processing speed and efficiency. Typically, DC starts from a centrally managed data set spread across multiple machines, which is assumed to contain independent and identically distributed observations. Each machine is tasked to process a comparable quantity of data. In contrast, clients independently join FL with their locally held data, which may vary significantly in quantity and distribution. While sharing some techniques with FL, distributed computing aims for computational efficiency and lacks its privacy focus.

On the other hand, meta-analyses (18) aggregate results across previously completed studies using statistical methods to account for their variations, thus allowing researchers to synthesise findings without accessing personal data and preserve the privacy of individual data sets. Here, FL collaboratively trains a joint model using distributed data to iteratively update it while meta-analysis constructs it in a single step from the pre-existing results. Multiple studies on sequencing data have demonstrated that FL produces results closer to centralised analysis than from meta-analysis (19, 20).

TREs (21) provide access to data within a controlled, secure computing environment for conducting analyses, almost always disallowing data sharing. Some TREs have a centralised data location and governance; an example is the Research Analysis Platform (RAP), the TRE for the UK Biobank [UKB; (22)]. Others, such as FEGA (23), are decentralised. Each institution maintains its data locally; only the relevant data are securely transferred to the computing environment when the analysis is authorised. Unlike FL, the learning process is not distributed across the data holders. Thus, the trade-off between TREs and FL is between a centralised, trusted entity with extensive computational facilities that can place substantial restrictions on the analysis, and a consortium that requires all parties to apply governance guidelines and provide compute, but can scale both data access and privacy guarantees.

### 2.1 Topologies

**Figure 2:**
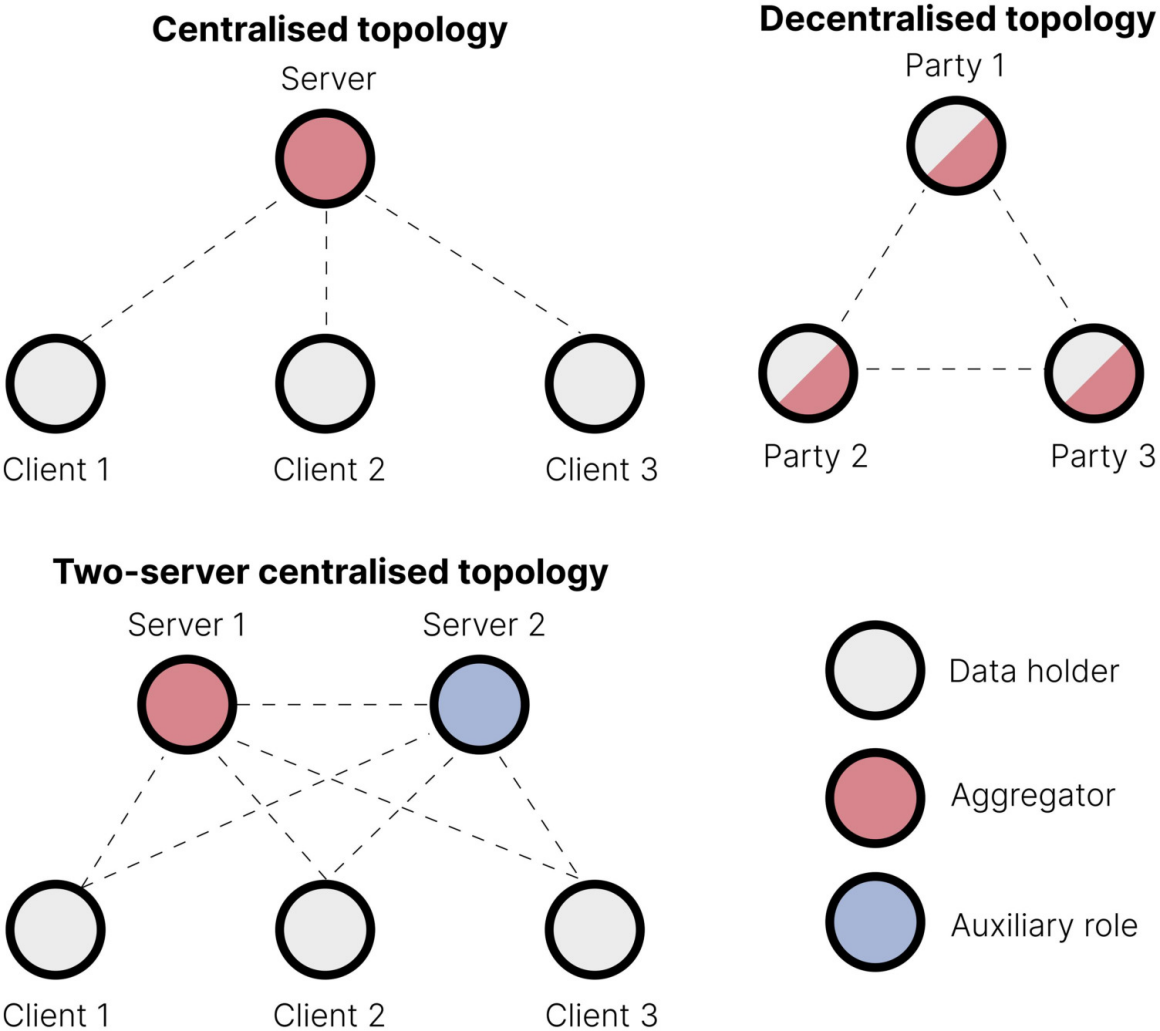
Different FL topologies. In centralised topologies, the data holders are typically referred to as *clients*, reflecting their interaction with a central server. In decentralised topologies, where no central entity exists, the participants are often called *parties*.

The *topology* of the FL consortium is determined by the number of participating parties and their defined interactions. Some examples are illustrated in Figure 2. The most common is the *centralised* topology, where multiple data-holding parties (the *clients*) collaboratively train a shared machine learning model through a central server (the *aggregator*) that iteratively collects model updates from each client, updates the global model, and redistributes it back to the clients. Typically, clients do not communicate directly; they only communicate with the central server. In contrast, a *decentralised* topology (24) lacks a dedicated aggregation server. All consortium parties can potentially serve as model trainers and aggregators, interacting through peer-to-peer communication. Hybrid configurations include, for instance, using two servers: one server handles aggregation of noisy local models, while the other performs auxiliary tasks, such as noise aggregation (25). Clients can communicate with the servers, and servers can communicate with each other, but clients cannot communicate with each other.

We will focus on the standard centralised topology and its two-server variant here because, to our knowledge, no bioinformatics applications use decentralised topologies.

### 2.2 Hardware and software

Hardware, software and models should be chosen with knowledge of the data and inputs from domain and machine learning specialists to design an effective machine learning pipeline (26).

In terms of infrastructure, FL requires computational resources for each client and server. The optimal hardware configuration depends on the models to be trained; at a minimum, each client must be able to produce model updates from local data, and each server must be able to aggregate those updates and manage the consortium. Connection bandwidth is not necessarily critical: to date, client-server communications contain only a few megabytes of data, reaching 150MB only for large computer vision models, and can be made more compact through compression and model quantisation (27). On the other hand, latency may be a bottleneck if it limits the hardware utilisation.

As for software, several dedicated FL frameworks, many of which are comparatively analysed in (28), provide structured tools and environments for developing, deploying, and managing federated machine learning models. While some frameworks, such as Tensorflow Federated [TFF; (29)], specialise in particular models, others support a broader range of approaches. Notable open-source examples include PySyft (30) and Flower (31). Both are supported by active communities and integrate with PyTorch to train complex models. PySyft is a multi-language library focusing on advanced privacy-preserving techniques, including differential privacy and homomorphic encryption. Flower is an FL framework: its modular design and ease of customisation make it particularly useful for large-scale and multi-omics studies involving heterogeneous devices and clients. We will provide examples using these frameworks in Section 3 before discussing frameworks explicitly designed for bioinformatics in Section 3.6.

Other frameworks target healthcare and biomedical applications, but not bioinformatics specifically. For instance, OpenFL (32) is designed to facilitate FL on sensitive EHRs and medical imaging data; it supports different data partitioning schemes (Section 2.4) but struggles with heterogeneous cross-device FL (Section 2.3). NVIDIA Clara, which was used in Dayan et al. (11), has similar limitations.

### 2.3 Usage scenarios: cross-device and cross-silo

FL applications take different forms in different domains. Many small, low-powered clients, such as wearable medical devices from the Internet of Things, may produce the data needed to train the federated machine learning model. Such *cross-device* communications are often unreliable: passing lightweight model updates instead of individual data largely addresses connectivity issues and privacy risks.

FL may also involve a small number of parties, each possessing large amounts of sensitive data (33), stored within their “data silos”. This setting, often called the emphcross-silo scenario, is common in healthcare and bioinformatics. Here, the main priority is to minimise the privacy risks associated with data sharing and comply with regulations. Additionally, minimising large data transfers is also computationally advantageous when modelling large volumes of information, such as whole-genome sequences.

These two scenarios differ in how they handle model updates. In the cross-silo scenario, all (few) data holders in the consortium must participate in each update. In contrast, we can rely on a subset of (the many) data holders in the cross-device scenario because each holds a smaller share of the overall data. This article focuses on the cross-silo scenario, as nearly all bioinformatics applications fall within this framework.

### 2.4 Data partitioning and heterogeneity

**Figure 3:**
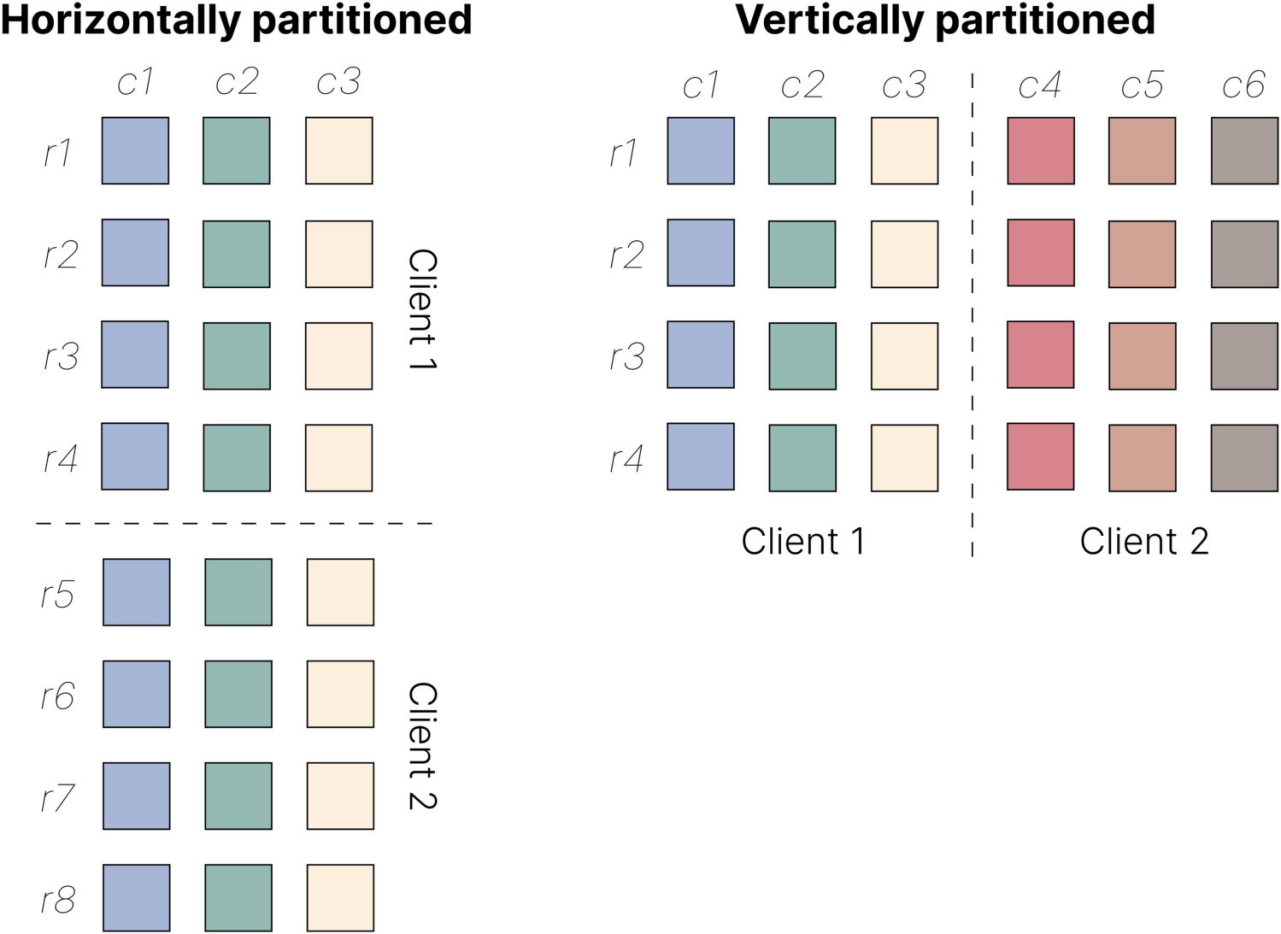
Horizontal and vertical data partitioning in FL. In horizontal FL (left), clients hold data sets with the same features (*c1–c3*) but different subsets of samples (*r1–r8*). In vertical FL (right), clients hold data sets with different features (*c1–c6*) but the same set of samples (*r1–r4*).

Data may be partitioned along two axes: each party may record the same features for different samples or features describing the same samples (Figure 3). In the first scenario, known as *horizontal* FL, different parties may each possess genomic sequencing data from different individuals. In contrast, in *vertical* FL, one party may hold data from one omic type (say, genomic data), while another may have data from a different phenotype or omic type (say, proteomic data) for the same individuals. Horizontal FL is by far the most prevalent approach in bioinformatics.

Significant variations in sample size and feature distributions between data holders often exist. This heterogeneity allows FL to better capture the variability of the underlying population, resulting in transferable models that generalise well (34). Clearly, if data holders collect observations from distinct populations, any federated model trained from them must be correctly specified to capture population structure and avoid bias in inference and prediction. If the populations are known, we can train targeted population-specific models alongside the global one (35). Otherwise, we can use clustering to identify them from the available data (36). Accounting for variations in measurements, definitions and distributions to harmonise data across parties is also fundamental but is much more challenging because access to data is restricted, even more so than in meta-analysis (27).

### 2.5 Security and privacy

FL reduces some privacy and security risks by design by passing model updates between parties instead of centralising data in a single location. However, it does not eliminate them completely.

In terms of privacy, deep learning models are the most problematic in machine learning because of their ability to memorise training data. They leak individual observations during training [through model updates; (37)], after training [through their parameters; (38)] and during inference [membership attacks; (39, 40)]. More broadly, individual reidentification is an issue for genetic data (41) and all the models learned from them. For instance, (42) has demonstrated that it is possible to identify an individual from the linear model learned in an association study from just 25 genes. However, such works make unrealistic assumptions on the level of access to the models and the data (43): even basic infrastructure security measures and the distributed nature of the data will make such identification difficult under the best circumstances. The privacy-enhancing techniques discussed in Section 2.6 can make such efforts wholly impractical.

As for security, we must consider different *threat models*, understanding what information requires protection, their vulnerabilities, and how to mitigate or respond to threats. Internal and external threats to the consortium should be treated equally with *security in depth* design and implementation decisions that consider parties untrusted. Security threats, such as membership attacks and model inversion attacks (44), can originate equally from parties and external adversaries that seek to abuse the model inference capabilities to extract information about the data. On the other hand, adversarial attacks are more likely to originate from consortium parties that seek to introduce carefully crafted data or model updates into the training process to produce a global model with undesirable behaviour. Some examples are data poisoning (45), manipulation (46) and Byzantine attacks (47).

Encrypting communication channels, implementing strict authentication (to verify each party’s identity) and authorisation (to control which information and resources each party has access to or shares) schemes, and keeping comprehensive access logs for audit can secure any machine learning pipeline, including federated ones. Similarly, using an experiment tracking platform makes it possible to track data provenance, audit both the data and the training process and ensure the reproducibility of results (26). These measures must be complemented by federated models resistant to these threats at training and inference time, as thoroughly discussed in Yin et al. (48).

### 2.6 Privacy-enhancing techniques

**Figure 4:**
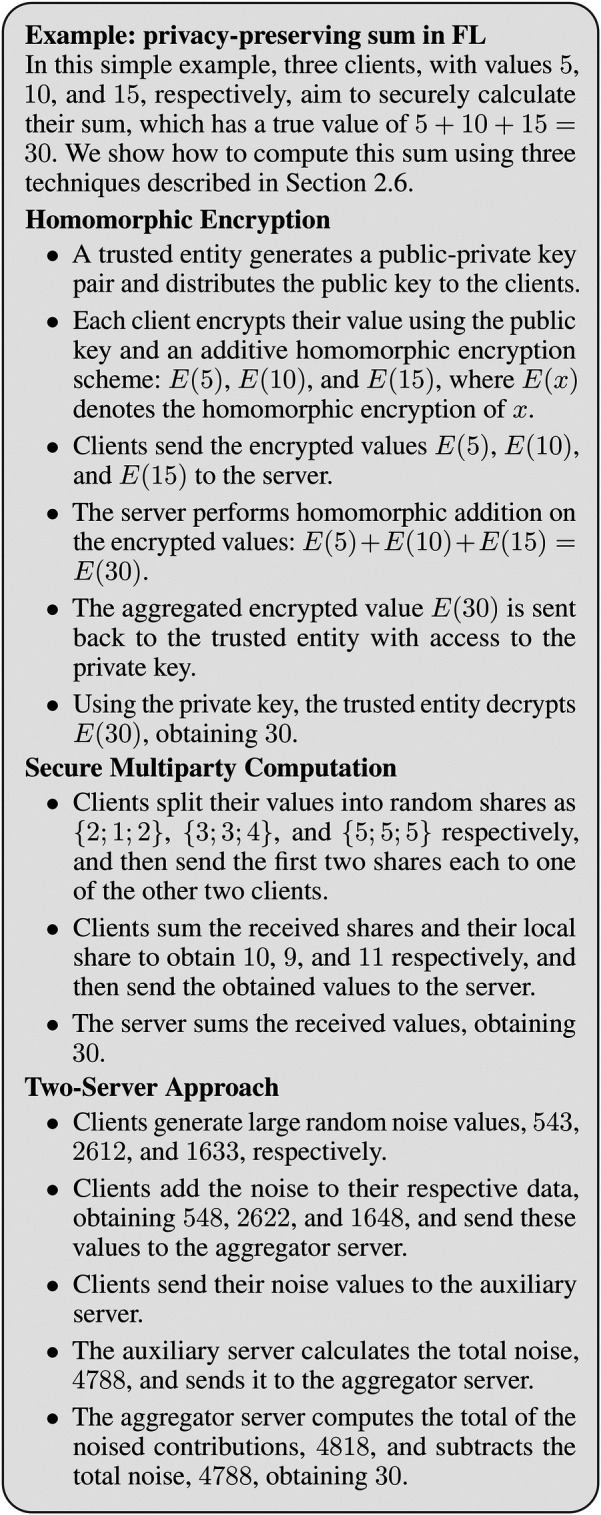
Example of privacy-preserving sum computation in FL using three different techniques. Note that although differential privacy is described in Section 2.6, it is not included in this example, as it would not be suitable for such a calculation.

Privacy-enhancing techniques improve the confidentiality of sensitive information during training. We summarise the most relevant below, illustrating them in Figure 4.

Homomorphic encryption [HE; (49)] is a cryptographic technique that enables computations to be performed directly on encrypted data (ciphertexts) without requiring decryption. The outcome of operations on ciphertexts matches the result of performing the same operations on the corresponding non-encrypted values (plaintexts) when decrypted. HE can be either *fully homomorphic* (FHE), which allows for arbitrary computations, or *partially homomorphic* (PHE), which supports only a specific subset of mathematical operations. For instance, the Paillier PHE scheme (50) only supports additive operations on encrypted data. FHE requires considerable computational resources for encryption and decryption. PHE is less flexible but computationally more efficient, making it a common choice in practical applications.

Secure multiparty computation [SMPC; (51)] is a peer-to-peer protocol allowing multiple parties to compute a function over their data collaboratively, similarly to Figure 2 (centre). Each data holder divides their data into random shares and distributes them among all parties in the consortium, thus ensuring that no single party can access the complete data set. The shares are then combined during the computation process, often with the assistance of a server, to produce the correct result while preserving data privacy. SMPC ensures high security with exact results and keeps data private throughout the computation process. However, SMPC is computationally intensive and requires peer-to-peer communication, leading to high communication overhead. Its complexity also increases with the number of participants, limiting scalability.

Another approach to securing FL is using an aggregator and a compensator server in a centralised two-server topology [Figure 2, right; (25)]. Each client adds a noise pattern to their local data, sharing the former with the compensator (which aggregates all noise patterns) and the latter with the aggregator (which aggregates the noisy data and trains the model). The aggregator then obtains the overall noise pattern from the compensator and removes it from the aggregated noisy data, allowing for denoised model training. This two-server approach is efficient: it requires neither extensive computation in the clients nor peer-to-peer communication. However, it makes infrastructure more complex and requires trust in both servers not to collude to compromise the privacy of individual contributions.

Unlike the above methods, which are encryption-based methods ensuring data confidentiality during transmission or storage, differential privacy, another popular technique for data-protection in federated learning, is not an encryption system but rather a technique that focuses on privacy by ensuring that the output of data analysis does not leak sensitive information about the underlying dataset. Differential privacy [DP; (52)] achieves this through a mathematical framework designed to ensure analyses remain statistically consistent, regardless of whether any specific individual’s data is included or excluded. This property guarantees that sensitive information about individuals cannot be inferred from the results up to a preset “privacy budget” worth of operations. DP is typically implemented by introducing noise into the data (53, 54), weight clipping in the training process (55, 56) or predictions (57, 58) to obfuscate individual contributions. The amount of noise must be carefully calibrated to balance predictive accuracy and privacy within the analysis: too little noise undermines privacy, and too much reduces performance. This effect is more pronounced within specific subgroups underrepresented in the training set (59).

## 3 Federated learning in bioinformatics

Most FL literature focuses on general algorithms and is motivated by applications other than bioinformatics, such as digital twins for smart cities (60), smart industry (61) and open banking and finance (62). Even the clinical literature mainly focuses on different types of data and issues (11, 63). Here, we highlight and discuss notable examples of FL designed specifically for bioinformatics, summarised in Table 2. They are all in the early stages of development, so their reliability, reproducibility, and scalability are open questions. However, they hint at the potential of FL to perform better than meta-analysis and single-client analyses on real-world data, comparing favourably to centralised data analyses where data are pooled in a central location while addressing data sharing and use concerns (15, 20).

**Table 2:** Summary of key federated learning applications in bioinformatics.

Field	Application	FL methods	Data	References
Proteomics and differential gene expression	Variance estimation, gene expression, cell type classification	FedDEqMS, FedProt, HyFed with limma voom, DL with Flower and TFF	Mass spectrometry, RNA-seq, 1–10 k individuals and 10–100 M biomarkers	(20, 25, 67, 70)
GWAS	Association testing, scalable regression	FedGLMM, federated GRM estimator, FedGMMAT, REGENIE with MPC/HE	SNPs, genotype and phenotype data, 2.5–275 k individuals and 0.5–38 M SNPs	(71–74)
Single-cell RNA-seq	Cell type classification	scFed: ACTINN, SVM, XGBoost, GeneFormer	scRNA-seq from 2–55 k cells and 1–2 k genes	(76, 77)
Multi-omics	Prognosis (cancer), diagnostics (Parkinson’s)	Vertical FL, adaptive neural networks, benchmarking with Flower	Genomics, transcriptomics, proteomics, 100–1,200 individuals and 100–700 features	(78, 79)
Medical Imaging	Classification, segmentation, semi-supervised training	Federated labelling, harmonised feature learning	MRI, x-rays, histology images, 5–71 k scans	(80–85)
Specialised tools	FL software for bioinformatics workflows	sfkit, FeatureCloud	All the data above	(19, 86–88)

### 3.1 Proteomics and differential gene expression

Proteomics studies the complex protein dynamics that govern cellular processes and their interplay with physiological and pathological states, such as cancer (64), to improve risk assessment, treatment selection and patient monitoring. Differential expression analyses focus specifically on comparing expression levels across different conditions, tissues, or cell types to identify genes with statistically significant differences (65).

In addition to the issues discussed in Section 2, FL in proteomics must overcome the challenge of integrating data from different platforms (66) while accounting for imbalanced samples and batch effects. Cai et al. (67) produced a federated implementation of DEqMS [FedProt; (68)] for variance estimation in mass spectrometry-based data that successfully identifies top differentially-abundant proteins in two real-world data sets using label-free quantification and tandem mass tags.

Zolotareva et al. (20) implemented a federated *limma voom* pipeline (69) on top of HyFed (25), which uses the aggregator-compensator two-server topology we described earlier. This approach was demonstrated on two extensive RNA-seq data sets, proving robust to heterogeneity across clients and batch effects. Hannemann et al. (70) trained a federated deep-learning model for cell type classification using both Flower and TFF and different architectures, with similar results.

### 3.2 Genome-wide association studies

Genome-wide association studies (GWAS) aim to identify genomic variants statistically associated with a qualitative (say, a case-control label) or quantitative trait (say, body mass index). These studies mainly use regression models, which can be largely trained using general-purpose federated regression implementations with minor modifications to address scalability and correct for population structure [see, for instance, (43)].

Li et al. (71) has developed the most complete adaptation of these models to federated GWAS in the literature: it provides linear and logistic regressions with fixed and random effects and accounts for population structure via a genomic relatedness matrix. Wang et al. (72) further provides a federated estimator for the genomic relatedness matrix. Finally, Li et al. (73) describes the federated association tests for the genomic variants associated with this model. All these steps incorporate HE to ensure privacy in the GWAS.

As an alternative, Cho et al. (74) built on REGENIE (75) to avoid using a genomic relatedness matrix and increase the scalability of GWAS while using MPC and HE to secure the data. Despite the overhead introduced by the encryption, this approach is efficient enough to work on a cohort of 401 k individuals from the UK Biobank and 90 million single-nucleotide polymorphisms (SNPs) in less than 5 h.

### 3.3 Single-cell RNA sequencing

Single-cell RNA sequencing (scRNA-seq) measures gene expression at the cellular level, rather than aggregating it at the tissue level as in bulk RNA sequencing, and identifies the distinct expression profiles of individual cell populations within tissues (89, 90).

Wang et al. (76) developed scFed, a unified FL framework integrating four algorithms for cell type classification from scRNA-seq data: the ACTINN neural network (91), explicitly designed for this task; a linear support vector machine; XGBoost based on Li et al. (77); and the GeneFormer transformer (92). They evaluated scFed on eight data sets evenly distributed among 2–20 clients, suggesting that the federated approach has a predictive accuracy comparable to that obtained by pooling the data and better than that in individual clients. However, the overhead during training increases with the number of clients, limiting the scalability to larger consortia. More recently, Bakhtiari et al. (93) introduced FedscGen, a federated implementation of scGen (94), a variational autoencoder-based method for batch effect correction. FedscGen employs secure SMPC for privacy-preserving aggregation and achieves results that closely match those obtained under centralised training.

### 3.4 Multi-omics

Proteomics, genomics, and transcriptomics capture different aspects of biological processes. Integrating large data sets from different omics offers deeper insights into their underlying mechanisms (95). Vertical FL allows multiple parties to combine various features of the same patients into multimodal omics data sets without exposing sensitive information (96). For instance, Wang et al. (78) trained a deep neural network with an adaptive optimisation module for cancer prognosis evaluation from multi-omics data. The neural network performs feature selection while the adaptive optimisation module prevents overfitting, a common issue in small high-dimensional samples (97). This method performs better than a single-omic analysis, but the improvement in predictive accuracy is strongly model-dependent. Another example is Danek et al. (79), who built a diagnostic model for Parkinson’s disease: they provided a reproducible setup for evaluating several multi-omics models trained on pre-processed, harmonised and artificially horizontally federated data using Flower. Their study identifies a general but not substantial reduction in FL performance compared to centrally trained models, which increases with the number of clients and is variably affected by client heterogeneity.

### 3.5 Medical imaging

Medical imaging studies the human body’s interior to diagnose abnormalities in its anatomy and physiology from digital images such as those obtained by radiography, magnetic resonance and ultrasound devices (98). It is the most common application of FL in the medical literature (16). As a result, protocols for image segmentation and diagnostic prediction are well documented. Notable case studies target breast cancer (10), melanomas (83), cardiovascular disease (84), COVID-19 (11, 85).

Machine learning applications that use medical imaging data typically face challenges, including incomplete or inaccurate labelling and the normalisation of images from different scanners and different protocols. Bdair et al. (80) explored a federated labelling scheme in which clients produced ground-truth labels for skin lesions in a privacy-preserving manner, improving classification accuracy. Yan et al. (81) also proposed an efficient scheme to use data sets mainly comprising unlabelled images, focusing on chest x-rays. Furthermore, Jiang et al. (82) apply FL to learn a harmonised feature set from heterogeneous medical images, improving both the classification and segmentation of histology and MRI scans.

### 3.6 Ready-to-use FL tools for bioinformatics

The need for user-friendly FL implementations of common bioinformatics workflows has driven the creation of secure collaborative analysis tools (87, 88, 99). Two notable examples are sfkit and FeatureCloud.

The sfkit framework (19) facilitates federated genomic analyses by implementing GWAS, principal component analysis (PCA), genetic relatedness and a modular architecture to complement them as needed. It provides a web interface featuring a project bulletin board, chat functions, study parameter configurations and results sharing. State-of-the-art cryptographic tools for privacy preservation based on SMPC and HE ensure data protection (100).

FeatureCloud (86) is an integrated solution that enables end users without programming experience to build custom workflows. It provides modules to run on the clients and servers in the consortium. Unlike sfkit, FeatureCloud allows users to publish applications in its app store, including regression models, random forests and neural networks. Developers must also document how privacy guarantees are implemented in their apps.

## 4 Practical insights on federation

This section offers practical insights to help readers interested in building a federated and secure analogue of an existing bioinformatics algorithm. We focus on horizontal FL with the centralised topology from Figure 2 (left). Consider K different clients, each possessing a local data set $X^{k}$, where k=1,…,K. Each data set contains $n^{k}$ samples, denoted as $x_{ij}^{k}$, where $i=1,\ldots ,n^{k}$ represents the sample index, and j=1,…,P represents the P features for each sample. We denote a row (column) of the matrix $X^{k}$ as $x_{i*}^{k}$ ($x_{*j}^{k}$). This describes a distributed data set of $N=\sum_{k=1}^{K}n^{k}$ observations:

$$X=\left[\begin{array}{c} X^{1}\\ X^{2}\\ \cdots \\ X^{K} \end{array}\right].$$

The following sections assume that an FL consortium has been established, the necessary infrastructure is operational, and an appropriate FL framework has been selected and installed. It is also assumed that a secure aggregation protocol has been chosen, such as those described in Section 2.6 and Figure 4. The choice of a specific secure aggregation protocol may depend on several factors, including technology and infrastructure (e.g., the availability of a particular FL topology that drives the choice), as well as privacy risks and scalability concerns, as discussed in Section 2.6. In the following sections, we provide a general overview of sum-based mathematical operations built upon a secure aggregation protocol, as well as operations involving federated averaging [FedAvg; (6)].

Coding examples using Flower (31) are available in our GitHub repository (https://github.com/IDSIA/FL-Bioinformatics). We chose Flower because it has a shallow learning curve for new FL users and provides a good balance between simplicity and flexibility when implementing custom algorithms. Riedel et al. (28) also identified Flower as a promising framework because it has a large, active, and growing community of developers and scientists, as well as extensive tutorials and documentation. In our examples, secure summation is performed using the secure aggregation protocol SecAgg+ (101). This protocol combines encryption with SMPC, using a multiparty approach in which each client interacts with only a subset of the others. It is particularly suitable for several FL contexts, as it is robust to client dropout and highly scalable. In particular, a relevant aspect of the bioinformatics domain is that it scales linearly with the size of the vectors to be aggregated (102).

### 4.1 Sum-based computations

Let $a^{k}$ be real numbers stored by individual clients. We define the secure sum of these numbers, performed through the selected secure aggregation protocol, as ${⊕}_{k=1}^{K}a^{k}$. We can build on this simple, secure sum to construct a wide range of operations. However, note that as the complexity of operations increases, the amount of information revealed to the server may also increase. Sum-based operations include:

- The *overall sample size* of the distributed data set as $N={⊕}_{i=1}^{K}n^{k}$ from the local sample sizes $n^{k}$.
- The *mean value of the* j*th feature*, given N, as
  $$M_{j}=\frac{1}{N}{⊕}_{i=1}^{K}\left[\sum_{i=1}^{n_{k}}x_{ij}^{k}\right].$$
  Each client computes the inner sum on their local data, whereas the outer one is a secure sum aggregated across clients by the server.
- The *variance of the* j*th feature*, given N and $M_{j}$, as
  $$V_{j}=\frac{1}{N-1}{⊕}_{k=1}^{K}\left[\sum_{i=1}^{n_{k}}(x_{ij}^{k}-M_{j}{)}^{2}\right],$$
  which can be used to standardise the jth feature as $(x_{*j}^{k}-M_{j})/\sqrt{V_{j}}$.
- The *Pearson correlation coefficient* of two features j and $j^{\prime }$, given $M_{j}$ and $M_{\;j^{\prime }}$, as
  $$\rho_{\;j,j^{\prime }}=\frac{\frac{1}{N-1}{⊕}_{k=1}^{K}\sum_{i=1}^{n_{k}}(x_{ij}^{k}-M_{j})(x_{ij^{\prime }}^{k}-M_{\;j^{\prime }})}{\sqrt{V_{j}V_{\;j^{\prime }}}}.$$
- The *matrix $X^{T}X$*, as $X^{T}X={⊕}_{k=1}^{K}(X^{k}{)}^{T}X^{k}$, where ⊕ is a secure element-wise sum. This matrix is equivalent to the covariance matrix for standardised data sets and is commonly used for PCA.

Beyond these general-purpose examples, many operations specific to bioinformatics pipelines also rely on simple sums. These operations are often straightforward generalisations or compositions of the examples introduced above.

In differential gene expression studies, for instance, filtering out weakly expressed genes is standard practice. Weakly expressed genes can be defined as those whose expression values fall below a specified threshold t in, for instance, 70% of the samples. Let $v^{k}$ be a vector belonging to client k, where each vector component represents the number of samples in which the expression level of the gene (e.g., counts) exceeds the threshold t. The server can securely calculate $v=\frac{1}{N}{⊕}_{k=1}^{K}v^{k}$ and identify weakly expressed genes as those whose corresponding components of v are smaller than 0.7.

A fundamental preliminary step in a GWAS is identifying the minor allele and its frequency. Let $a^{k}$, $c^{k}$, $g^{k}$, and $t^{k}$ be vectors belonging to client k, where each component corresponds to a specific SNP. The components of $a^{k}$, $c^{k}$, $g^{k}$, $t^{k}$ represent the number of samples in which nucleotides A, C, G, T are observed, respectively. The server can securely compute the aggregated allele counts across all clients as $a={⊕}_{k=1}^{K}a^{k}$ and similarly c, g, t (where t can also be computed by difference from N and the other three vectors). For each SNP, the minor allele is determined by comparing the corresponding components of a, c, g, t: the allele with the smaller value is designated as the minor allele. This operation is crucial because the minor allele within a single client’s population may differ from the minor allele when considering the whole distributed data set. Ensuring a consistent definition of the minor allele across all clients is essential for reliable downstream analyses.

### 4.2 Federated averaging computations

FedAvg is a widely used algorithm for training deep neural networks in FL. It iteratively computes a weighted average of model parameters across clients, with weights proportional to the local sample sizes $n^{k}$. Thus, it can be applied to any parametric model, including linear models.

FedAvg proceeds as illustrated in Figure 1. The server first broadcasts an initial global model with parameters $w_{0}$. At each step of the algorithm, clients start with the global model $w_{t}$ and perform local updates to produce updated local models $w_{t+1}^{k}$. The global model is updated after each round of local training as the weighted sum of the local models:

$$w_{t+1}={⊕}_{k=1}^{K}\frac{n^{k}}{N}w_{t+1}^{k},$$

where we use the secure sum ⊕ for aggregation (FedAvg is itself a sum-based operation). After aggregation, the updated global model is distributed back to the clients.

However, many bioinformatics pipelines rely on linear models rather than deep learning models. One commonly used model is logistic regression, which is applied in tasks such as gene expression analysis and GWAS. A federated implementation of logistic regression can be achieved by starting with a standard implementation and applying FedAvg, which aggregates the local models after a specified number of iterations performed by the local logistic regressions.

## 5 Legal aspects of federated learning

The legal frameworks used within FL consortia are rarely discussed in the literature. Ballhausen et al. (103) describes both the technical and legal aspects of a European pilot study implementing a federated statistical analysis by secure multiparty computation. They established agreements between parties similar to those between participants in a multi-centre clinical trial, as using SMPC and exchanging model gradients was legally considered data pseudonymisation (rather than anonymisation). FL was determined to require the same level of data protection as regular data sharing, which is also the most conservative course of action suggested in Truong et al. (9) and Lieftink et al. (104). All clients jointly controlled the consortium and were responsible for determining the purpose and means of processing, including obtaining approval from the respective Ethics Committees. Sun et al. (105) similarly describes the server in their consortium as a trusted and secure environment, supported by a legal joint controller agreement between the data owners.

Following Ballhausen et al. (103), establishing an FL consortium could be expected to require all participating and involved parties to execute agreements that regulate their interactions, the so-called DPA or DSA (data protection/processing/sharing agreements). Doing so will establish the level of trust between parties and their responsibilities towards each other, third parties, and patients. Risk aversion suggests that it should include a data-sharing clause to allow for the sharing of information, similar to a centralised analysis, as described in Figure 1. No party has access to the data of other parties. Still, it is theoretically possible that, in some cases, the model updates shared during FL could be deanonymised by malicious internal or external attackers (9). Parties may then be reluctant to treat that information as non-personally identifiable without formal mathematical proof of anonymisation and prefer to establish data protection responsibilities with a data-sharing agreement. In the EU (GDPR), but also other jurisdictions (national data protection laws), “all the means reasonably likely to be used should be considered to determine whether a natural person is identifiable” (1). Securing infrastructure in depth using best practices from information technology, defensive software engineering, and data by secure computing and encryption can make malicious attacks impractical with current technologies [security by design and by default; (106, 107)]. In addition, when FL involves models other than deep neural networks, if the contributions of individual parties are well balanced across the consortium and include a sufficiently large number of individuals, the information exchanged may very well be the same summary information routinely published as supplementary material to academic journal publications (108). A recent systematic literature review of privacy attacks in FL has also highlighted that many of them are only feasible under unrealistic assumptions (8). Therefore, reducing the amount of information shared during FL and using secure computing must be considered to provide increased protection against data leaks and misuse. Advertising such measures as a key feature of the FL consortium will make partners and patients more comfortable with contributing to federated studies [see, for instance, (103)]. Lieftink et al. (104), which investigated how FL aligns with GDPR in public health, also acknowledges that FL mitigates many privacy risks by enforcing purpose limitation, data use and information exchange minimisation, integrity and confidentiality (at a cost, as discussed in Section 2.4).

Furthermore, consortium parties must agree on how to assign intellectual property (IP) rights. Bioinformatics research often has practical applications in industry, which may involve patenting the results and apportioning any financial gains arising from their use. Parties in the consortium jointly control it and should share any gains from it (109). FL consortia are no different in this respect. From a technical standpoint, watermarking techniques for tracking data provenance and plagiarism have been adapted to FL (110) to identify data and model theft.

Additionally, the agreement establishing the FL consortium must outline its relationships with third parties and their corresponding legal obligations. Third parties that have access to the infrastructure may be required to sign a data processing agreement to guarantee the safety and privacy of data. In many countries in Europe, as well as in the US, patients have the right to withdraw their consent to use their data at any time. This, in turn, may require implementing procedures to remove individual data points from future federated analyses.

We summarised these considerations in Table 1, along with the key differences from the alternatives we discussed in Section 2. FL provides increased protection against data and model leaks, which should reduce the perceived risk for parties and patients in contributing to the consortium. However, out of an abundance of caution, establishing a consortium-wide data-sharing agreement may help allocate and reduce party responsibilities in the event of a privacy breach. The use of FL has a limited impact on other legal aspects of collaborative analysis, such as IP handling and requirements for third parties, because it is a technical solution that does not change the fundamental legal rights and responsibilities of the parties involved in the consortium. Table 3 summarises the key legal and procedural steps required to implement federated learning in biomedical research, as detailed above.

**Table 3:** Overview of legal, procedural, and technical actions in federated learning, with relevance to governance, data, and software.

Action	Documentation	Data controllers responsabilities	Data processors responsibilities	Notes
Ethical approval	Study protocol (including data sharing information).	Obtain approval from local ethics committees.	–	Making the use of secure computing transparent (thus limiting the ways data can be processed) should support ethics committees’ trust in the approach.
Consortium setup	Cooperation/data protection/processing/sharing agreements	Joint controllers, responsible for the purposes and means of processing and for data security and purpose adherence.	Contractual responsibilities stemming from controllers’ data security and purpose adherence responsibilities.	Although parties cannot access each other’s data, agreements often permit the sharing of private information to address potential leakages from shared model parameters.
Data protection officer (DPO) advice	GDPR-compliant documentation, Data Protection Impact Assessment (DPIA), software documentation.	Appoint DPO if needed under GDPR (Article 35) and obtain advice.	–	Consortium agreements should specify how DPO responsibilities are allocated; a single DPO may be designated at the consortium level or appointed from a subset of partners.
Clinical data collection (retrospective or prospective)	Informed consent forms, data collection protocols.	Collect consent from all patients. Guarantee the right of withdrawal.	–	Data should generally be considered pseudo-anonymised. Explaining secure computing methods to patients should foster trust and informed participation.
Code deployment	Software license, deployment agreements.	Grant local software deployment compliant with deployment agreements.	Guarantee the software and platform’s security and usage comply with the purposes and licences.	The consortium should agree on where deployment happens (e.g., trusted execution environment - TEE), what is deployed and how deployments are authorised.
Intellectual property (IP)	IP ownership agreements, licensing terms, publication policies.	If the trained model is protected by IP rights, its ownership and usage are governed by the terms of the contract.	Do not automatically participate in IP generated from the trained model.	In FL, the trained global model is typically viewed as a joint IP artefact. There is ongoing work to establish IP allocation models, licensing templates, and tailored governance mechanisms.
Model governance	Model versioning logs, audit trails, validation reports, access control policies.	Guarantee model trustworthiness	Facilitate model governance and auditability	While stringent requirements are requested only in case of model deployment, analytical or research-only contexts still require principled governance to ensure reproducibility, accountability, and ethical compliance.

We now proceed to discuss how FL facilitates compliance with the key requirements of GDPR (1) through its architecture. Data providers, which have a complete control over and a more intimate knowledge of the data they collected as well as a direct connection to data subjects, act in a “data controller” role (Articles 4 and 24), taking “appropriate technical and organisational measures” (Article 25) to ensure privacy and security, thus minimising the risk of data breaches. Therefore, they can directly scrutinise their use, notify data subjects about it to request consent (Article 9); allow them to withdraw their data (Article 7); ensure lawful, fair and transparent processing (Articles 12–15); and directly assess risks to data subjects and minimise them through appropriate legal agreements. Consortium parties, which include both data controllers (as clients) and data processors (as servers, compute facilities, as defined in Article 4), are also required to use the techniques described in Section 2.5 to ensure data security and privacy beyond what is provided by base FL (privacy by design, Articles 24, 25 and 32). For the same reasons, FL facilitates compliance with the EU AI Act (2). Some of its requirements strengthen those in the GDPR, such as data minimisation, localisation, transparency, auditability, security, and data quality. Additionally, the EU AI Act requires efforts to mitigate bias, ensure the robustness of models, implement human oversight, and assess high-risk systems. Data controllers are in the best position to ensure these requirements are met. Collectively, they can provide more representative samples that are less prone to bias and fairness issues. Finally, the presence of multiple data controllers in the consortium also implies that these requirements are verified by several independent parties.

In contrast, the US AI Act is more flexible in its requirements, which are left to sector-specific agencies to define and enforce. It focuses more on party self-regulation and harm remediation rather than universal legal mandates and prevention. As a result, cross-border EU-US consortia should rely on the EU-US Data Protection Framework (111) or put in additional safeguards as required by the GDPR (Article 46). Even so, FL’s privacy stance naturally fits well with the practical implementation of the US AI Act.

## 6 Challenges and future directions

Federated learning (FL) has evolved rapidly over its relatively short lifetime, becoming a widely adopted methodology across diverse domains. In a recent article (112), which includes several authors of the seminal FL paper (6), the progress of the field is reviewed, and key challenges for its future development are outlined. The authors propose a refined definition of FL centred on privacy principles and analyse how core concepts such as data minimisation, anonymisation, transparency and control, verifiability, and auditability have evolved and are expected to play a major role in the future. They identify three primary challenges for the field: scaling FL to support large and multimodal models, overcoming operational difficulties arising from device heterogeneity and synchronisation constraints (particularly relevant in cross-device FL), and addressing the current lack of verifiability in deployed systems. As a potential means to address this latter aspect, the authors highlight trusted execution environments (TEEs) (113) as a promising technology. A TEE is a secure area within a processor that executes code and processes data in isolation from other software, protecting it from tampering or unauthorised access even if the main operating system is compromised.

Complementing these insights, other surveys examine additional aspects of the anticipated future developments in federated learning (FL) research. Wen et al. (114) call for more efficient encryption schemes and greater overall efficiency in FL, including strategies to reduce communication costs between clients and servers. They also emphasise the importance of novel aggregation strategies that can better handle client heterogeneity. In the context of multimodal FL, aggregation must often reconcile contributions when clients supply only partially overlapping modalities, which represents an additional complexity beyond standard heterogeneity. Yurdem et al. (115) highlight the emerging paradigm of FL as a Service, in which federated learning training is conducted through ready-to-use platforms such as FeatureCloud (86) and sfkit (19), discussed in Section 3.6. This approach allows institutions to participate in collaborative model development with minimal software and deployment effort, thereby simplifying implementation and facilitating cross-organisational collaboration. Looking ahead, the emergence of federated foundation models is expected to define the next phase of research in this field. Their development will require progress across several key dimensions, including improving efficiency through advanced aggregation methods and optimised computational and communication frameworks; strengthening trustworthiness by increasing robustness to attacks; and enhancing incentive mechanisms that reward clients according to the quality of the models provided. Collectively, these advances are expected to be essential for making large-scale federated models practical and scalable while maintaining manageable communication costs (116).

The prospects of federated learning in bioinformatics depend on how the legal and technical landscape will develop. Further legislation will progressively regulate the use of machine learning and AI models, defining and restricting how data can be shared and used. As its effects percolate through protocol and product development, many aspects of federated learning will likely take a more definite shape.

Firstly, the security and privacy risks are likely to become more clearly defined. Jurisprudence will naturally shift from general guiding principles, such as the EU AI Act, to practical compliance rule sets as products based on federated learning enter the market. How to assess sensitive aspects of data (re)use will also likely be standardised (117), using the vast collection of available data and model cards as a starting point (118, 119). What threat models are relevant, what security measures are appropriate at the infrastructure level, and what attacks are feasible will then become clear, possibly confirming the irrelevance of many that have been speculated in the literature (43). The evolution of encryption and differential privacy techniques may also allow different types of data to be treated as anonymised, depending on their nature and the theoretical guarantees of those techniques. Some data (say, single-cell transcriptomics) are intrinsically more difficult to tie to a specific individual than others (say, whole genome sequences), and different privacy-enhancing techniques are more effective than others at mitigating various types of risk.

Secondly, the evolution of data and models will require periodic reevaluation of the trade-offs discussed in this paper. The ever-increasing volumes of data used to train federated models will eventually force a cost-benefit analysis for resource-intensive techniques like HE and MPC; lighter approaches may be the only feasible choice at scale, provided regulations permit their use. Modelling approaches will undergo a similar selection process, as they will be required to evolve to handle new biological and clinical data types in larger quantities and across multiple modalities. In this respect, we foresee that federated learning in bioinformatics may diverge from the mainstream, which has largely standardised on deep learning models (15, 16). Historically, bioinformatics has produced distinct model classes for different types of data. Federating them, optimising them, and assessing their privacy risks will require significant research and engineering efforts before they are suitable for practical applications.

## 7 Conclusions

Independent research efforts in several bioinformatics domains have shown federated learning to be an effective tool to improve clinical discovery while minimising data sharing (10, 12, 13, among others). FL enables access to larger and more diverse data pools, resulting in faster and more robust exploration and interpretation of results. At the same time, it provides enhanced data privacy and can seamlessly incorporate advanced, encrypted and secure computation techniques. Despite the increased computational requirements and reduced ability to explore and troubleshoot issues with the data (104), the benefits of FL may outweigh these additional costs.

Therefore, federated learning can potentially mitigate the risks associated with national regulations if implemented in a manner that is secure by design and by default. Its use may also make patients and institutions more confident in participating in clinical studies by reducing privacy and data misuse risks. However, the reliable use of federated learning and its effective translation into clinical practice require a concerted effort by machine learning, clinical, and information technology specialists. All their skills are necessary to accurately evaluate the associated risks and expand its practical applications in bioinformatics beyond the early-stage applications reviewed in this paper.
